# A Case of Pulmonary Syphilis Resulting in Multi-organ Dysfunction and Consequential Demise of a 19-Year-old Male

**DOI:** 10.7759/cureus.5560

**Published:** 2019-09-03

**Authors:** Sonam Sharma, Aditya Mehta

**Affiliations:** 1 Internal Medicine, St. James School of Medicine, The Quarter, AIA; 2 Ophthalmology, San Antonio Military Medical Center, San Antonio, USA

**Keywords:** pulmonary syphilis, multiorgan dysfunction, immunosuppression

## Abstract

This case presents a young male, with a history of high-risk sexual behavior and immunosuppression, admitted with multi-organ dysfunction and pronounced dead within 24 hours of admission. After an extensive investigation, to include post-mortem studies, his symptoms were attributed to secondary syphilis, which was confirmed with high rapid plasma reagin (RPR) titers (1:128) and positive fluorescent treponemal antibody absorption test (FTA-ABS). The patient developed rapid hypoxemia and hemodynamic instability with pulseless electrical activity (PEA) arrest and was unable to be resuscitated after many rounds of cardiopulmonary resuscitation (CPR). An autopsy revealed syphilis spirochetes in the liver, heart, lungs, and brain.

## Introduction

Syphilis is a sexually transmitted disease caused by a spirochete *Treponema pallidum*. Syphilis can involve various organs in secondary and tertiary stages. Pulmonary involvement is rare. We present a case of a young male admitted with pulmonary syphilis resulting in multi-organ dysfunction and was pronounced dead within 24 hours of admission. An extensive investigation attributed his symptoms to secondary syphilis. The patient developed rapid hypoxemia and hemodynamic instability with pulseless electrical activity (PEA) arrest and was unable to be resuscitated after many rounds of cardiopulmonary resuscitation (CPR). 

## Case presentation

The patient is a 19-year-old bisexual male who presented to the ED with one week history of progressively worsening nonproductive cough and night sweats. The patient had dyspnea which lasted approximately 30 minutes, and which prompted his mother to bring him to the ED. The patient was recently diagnosed with right lung pneumonia and urethritis at an outside facility, thought to be secondary to gonorrhea and chlamydia, for which he was treated with one-time dose of intra-muscular ceftriaxone IM and a 10 day course of oral doxycycline and azithromycin that he was currently taking. 

Past medical history is notable for Bipolar Type I that initially manifested as erratic behavior and hypersexuality and nonspecified myositis. The patient’s current medications included aripiprazole, prednisone 12.5 mg daily, and methotrexate 14 mg weekly. The patient’s surgical history included a tonsillectomy and adenoidectomy. He had no allergies to drugs. He smoked marijuana occasionally but denied any alcohol or intravenous drug use. 

On examination, his temperature was 98.5°F, heart rate was 128 per minute and regular, blood pressure 110/76, and respiration 24 per minute. General examination showed a pale, young male without severe distress. His mental status appeared to be normal. He had conjunctival pallor but without icterus, adenopathy, meningismus, or any buccal lesions. A soft holosystolic murmur was appreciated at the cardiac apex; lung sounds were diminished in the right middle lobe. The abdomen was soft but mildly tender to palpation in all quadrants without rebound or guarding. A maculopapular rash was noted on the palmar surfaces of hands and soles of feet. External genitalia were without rashes. The head of the penis was not able to be visualized secondary to phimosis. 

Initial laboratory investigation showed a leukocytosis with white blood count of 15.2 with a manual diff indicating 82% neutrophilia. A renal function panel showed elevated anion gap metabolic acidosis with lactate of 6.7 mmol/L (Na, Cl, HCO3 15 mmol/L). Liver transaminases were elevated with alanine aminotransferase (ALT) 1700 and aspartate aminotransferase (AST) 1627. Total bilirubin was normal at 1.5 and alkaline phosphatase was 85 and INR of 2.2. A disseminated intravascular coagulation (DIC) panel indicated a mildly elevated D-dimer 11.94 ug/mL but without schistocytes. An AP portable chest X-ray showed multifocal opacities predominately in the right and left mid-lung and concerns for cardiomegaly without pleural effusions. A urinalysis was negative for nitrites and leukocyte esterase. An abdominal CT scan with IV contrast indicated hepatomegaly that was heterogeneously edematous. 

The patient was started on broad spectrum antibiotics to include piperacillin/tazobactam and vancomycin IV given a clinical picture of sepsis with pneumonia and concurrent immunosuppression. Ceftriaxone IV was also given due to concern for possible meningococcemia. A rapid plasma reagin (RPR), fluorescent treponemal antibody absorption test (FTA-ABS), HIV ELISA, and HIV RNA PCR were ordered. The results of the RPR were strongly positive (1:200) while the FTA-ABS was still pending. A repeat DIC panel revealed worsening elevation of PT/aPTT (27.5/33 sec), a dropping fibrinogen (179 mg/dL), and D-dimer of >20,000 ug/mL. The patient’s physical exam at that time did not reveal any petechia or purpura. 

Eighteen hours into the admission, the physician was alerted by the bedside nurse that the patient was in distress. At arrival to bedside, the patient was diaphoretic, tachypneic, complained of a new headache, and exhibited a worsening mental status. Pulse oximetry could not detect a level, but manual BP showed a systolic blood pressure (SBP) of 110. He then fluctuated between sinus tachycardia at 150 to bradycardia in the 40s. He was given atropine at that time for symptomatic bradycardia. Next, the patient began to posture with left upper extremity extension, right upper extremity flexion, and lower extremity extension. Shortly thereafter, his pulse was lost and PEA arrest was called. 

The patient was then coded for roughly one hour. He frequently achieved return of spontaneous circulation (ROSC), however, would only maintain a pulse for no more than one minute. He received many doses of epinephrine, bicarbonate, calcium gluconate, and dextrose. Epinephrine and dopamine drips were started. He received 23.5% saline out of concern for brain herniation. Bedside echo during the brief episodes of ROSC revealed depressed myocardial contractility, and no evidence of pneumothorax. Extracorporeal membrane oxygenation (ECMO) team was consulted, however, veno-arterial extracorporeal membrane oxygenation (VA ECMO) / extracorporeal cardiopulmonary resuscitation (ECPR) would require anticoagulation and with the concern for an intracranial catastrophe, ECMO was contraindicated. Nonetheless, after approximately 60 minutes of CPR, the patient developed asystole and despite further efforts, death was pronounced. 

## Discussion

This case presents a rapid evolution of multi-organ failure, in an immunocompromised patient, from secondary syphilis, to include neurosyphilis, thus ultimately leading to hypoxemia and subsequent PEA arrest. The incidence of syphilis has dramatically risen since the 1990s with 83% of new cases attributed to men who have sex with men [1]. Primary syphilis begins with a painless, penile chancre and if left untreated, progresses to secondary syphilis within three to six weeks. Immunocompromised patients, such as those with concomitant HIV or undergoing immunosuppressive therapy, are at an especially increased risk of presenting with systemic manifestations of secondary syphilis [1-3]. A study evaluating 11,135 individuals with HIV identified 151 individuals with active syphilis out of which maculopapular secondary syphilis was present in 29.1%, neurosyphilis in 16.6% and ulcerating secondary syphilis in 7.3% [4].

In our patient, sepsis from syphilitic pneumonia was high on the differential given his high-risk sexual behavior, recent history of urethritis and exam notable for diminished right sided air entry on auscultation in conjunction with a maculopapular rash on the palmar hands. His chest X-ray indicated bilateral multifocal opacities. Due to patient’s immunocompromised status, broad spectrum antibiotics with IV vancomycin and piperacillin/tazobactam, and ceftriaxone were initiated. 

A post-mortem broncheoalveolar lavage and pleural biopsy confirmed the presence of treponema pallidum. Pulmonary involvement in syphilis is rare and there have been 16 reported cases of pulmonary involvement dating back to 1966. In previous reports detailing pulmonary involvement, patients received either intramuscular (IM) or IV penicillin G with symptom resolution within 6-48 hours . This patient completed a seven-day course of doxycycline and continued with IV ceftriaxone therapy, which has been shown to be comparable or even more efficacious at treating syphilis [5-7]. It is possible that his pulmonary infiltration may have represented a nidus for further and rapid hematogenous spread of *Treponema pallidum*.

To further add to his multi-organ dysfunction, the patient may have also developed a Jarisch-Herxhiemer reaction, which is a severe inflammatory reaction mediated through an endotoxin-like release of lipoproteins from the dead bacteria [3]. This reaction can be observed in about 90% of patients with secondary syphilis. The patient’s coagulopathy in conjunction with his altered mental status and decorticate posturing raised suspicion for an intracranial bleed, however, post-mortem imaging and autopsy did not find any evidence. Ultimately, his volume overload with congestion of the hepatic veins and liver, coagulopathy in the setting of hepatitis, and failure to regulate autonomic tone, led to hypoxemia, PEA arrest, and his eventual death.

## Conclusions

Pulmonary involvement is a rare finding in secondary syphilis. However, men who have sex with men (MSM) patients with syphilis may have concurrent immunosuppression that may increase their risk of pulmonary involvement, thus leading to rapid hematogenous spread. Patients undergoing treatment for secondary syphilis have a high risk for developing a Jarisch-Herxhiemer reaction, and should be monitored closely. To our knowledge, this is the first case of pulmonary syphilis leading to rapid multi-organ failure and PEA arrest.
